# Interesting Account of the Various Forms of Scarlatina from the Same Source of Infection

**Published:** 1817-03

**Authors:** 

**Affiliations:** Zerbst.


THE LONDON
### Medical and Physical Journal.

3 OF VOL. XXXVII.] MARCH, 1817. [NO. 217.

"For many fortunate discoveries in medicine, and for the detection of nume-
"rous errors, the world is indebted to the rapid circulation of Monthly
"Journals; and there never existed any work to which the Faculty in
"EUROPE and AMERICA were under deeper obligations than to the
"Medical and Physical Journal of London, now forming a long, but an
"invaluable, series."—RUSH.

---

### *For the London Medical and Physical Journal.*

### *Interesting Account of the various Forms of Scarlatina from the same Source of Infection;* by Dr. HENNING, of Zerbst.

IN October last, I happened to receive a visit of a friend, who, for some time, had been sojourning in the atmosphere of scarlatinous patients. My own four children, the eldest of whom was then twelve, and the youngest seven years, and my maid servant, though susceptible, had hitherto remained free from infection. In the presence of my children, and whilst the servant was waiting on us, my friend told me, that, at the place of his residence, he had lately had several patients with scarlatina, some of whom were actually then lying in a very dangerous state; and confessed that within the last few days he had lost nine children with the cynanche maligna. This relation made his visit less agreeable to me at that moment than it would have been at any other time. Six days having elapsed, our servant first began to complain of a sore throat and difficulty of swallowing; but nothing was to be discovered by examining the mouth, on the palate, &c. My children gradually sickened in a similar manner. In the latter, the scarlatina broke out regularly, and normally run its course without particular symptoms of malignity. In the servant, on the contrary, not the least symptom of eruption could be discovered: she had fever, without the least remission; the pulse 120 in a minute, the pain in the throat becoming worse from one minute to the other, so that on the third day she was in danger of suffocation. From the first day I employed a surgeon for the treatment of the local complaint, who, by injections, vapours, leeches, vesicants, sinapisms, and embrocations, tried to counteract the danger. The girl not being capable of swallowing even a drop of Elder-tea, any application of internal medicine became utterly impracticable. The bowels were kept in regularity by daily glysters, and pediluvia applied. Notwithstanding the other dreadful symptoms, there was no trace of inflammation or swelling to be seen in the oral cavity, but externally the throat appeared swollen, and distended from the larynx downward, and felt very hot. Half an inch under the cartilago thyroideus she appeared to have the most pain, on touching the part where the leeches and poultices had been applied, and a mercurial ointment had been rubbed in. In this dangerous situation I prescribed a few grains of calomel, to be triturated with sugar, to be held in the mouth, and, there being suffered to melt, to swallow some of it, if possible. This, however, proved also abortive; and I was obliged to relinquish all further attempts of giving medicine internally, and make use of whatever external ones I could devise. The girl being very weak, and naturally of an asthenic constitution, nothing remained but to treat this inflammatory tate continually with external stimulants. I, indeed, suspected a metastasis of scarlatinous matter to the œsophagus, the more so as there was not the least cough to mark an irritated trachea. For this reason, I tried the more to obviate the urgent local inflammation and the pressing danger, and then waited for what would follow. Things remained in this horrible state full seven days, during which time she laboured under the greatest anxiety, and the sweat trickled down her face in large drops. At last, on the seventh day, the first symptoms of amendment began to shew themselves: the fever, which hitherto had been of an uninterrupted continuance, now shewed considerable remissions; the pain in the throat abated; the pulse became more moderate; and she was able to swallow. The anxiety subsided; sleep returned, and with it a salutiferous clammy sweat. The urine, which hitherto had been much concentrated, became thick, and had a brick-like sediment. A kind of salivation, of a tough and saltish matter, also took place. The head, which hitherto had ached most violently, now became easier; the swelling of the throat abated; and she also expressed a desire for nourishment. A dish of some gelatinous preparation of grits being prepared for her, she took a few tea-spoonsful. On the eighth day she felt a violent tickling in the throat, which constantly irritated her to cough, and which she only could overcome by swallowing some mucilage of gum arabic or liverwort. This state lasted until the twelfth day, during which time the menses returned. She now mended rapidly, and, with the cough, there appeared an expectoration of a membranaceous substance. She coughed up whole pieces of that sort; and this expectoration lasted uninterruptedly from eight to ten days. With it she felt, at the same time, a kind of jumping in her mouth, particularly when she was going to swallow; and this sensation only ceased, or became moderated, on swallowing some milk, or something cool. One morning she coughed up a piece of that sort more than two inches in length, which perfectly resembled the inner skin of an egg. This expectoration abated gradually; the cough left off, so that she recovered completely without taking the least internal medicine. A rigorous, yet nourishing, diet, moderate use of the free air, and a few mild purges, completed the cure.

The whole exanthema had, in this case, taken possession of the surface of the œsophagus, without even the mouth at all participating in it,—most likely on account that the too great vicinity of the gullet had served as a derivans. All that time the patient had been without either nourishment or refrigerant medicines. Seven days she had been in imminent danger of suffocation, yet without any suffering of the respiratory organs; and benignant Nature, supported by a few external stimulants alone, conquered a disorder in a weak asthenic habit, which, for a space of time, threatened every moment to prove fatal. This metastasis is undoubtedly a phenomenon, with which the practitioner meets but seldom. After the period of convalescence, her feet became œdematous, which gave way to a few doses of acetated ammonia. What, besides these, appeared most strange in this morbid state, was, that her whole body afterward began to scale off, though not the least exanthema had been observed, and though the whole of the internal surface of the œsophagus seemed to have come away by the egg-shell-like membranes that had been coughed up. After the expiration of six weeks, she was so well recovered as to be able to attend to her usual domestic duties as before.

In my youngest girl, a child of little more than nine years, the exanthema terminated in a nervous fever, accompanied with œdema, which, however, at last gave way to the most powerful stimulants, though a symptom ominous of nothing good had occurred for some days.—Such was the progress of the disorder in my family.

Lastly, my servant's sister, a married woman of this place, came to see her relation, bringing with her a child three years old. Both became infected by the disorder, and communicated it to her husband. All three were in the greatest danger. It was also remarkable that the woman had not the exanthema *properly*, but only pustulous open ulcers on the surface of her face; but the husband and the child were thickly covered with it all over;—but all the three, in particular the husband, suffered at the same time the most violent cynanche.

